# PECLIDES Neuro: A Personalisable Clinical Decision Support System for Neurological Diseases

**DOI:** 10.3389/frai.2020.00023

**Published:** 2020-04-21

**Authors:** Tamara T. Müller, Pietro Lio

**Affiliations:** Computer Laboratory, Department of Computer Science and Technology, University of Cambridge, Cambridge, United Kingdom

**Keywords:** decision support, random forest, precision medicine, neurological diseases, personalisable medicine, machine learning, Alzheimer's Disease, Parkinson's Disease

## Abstract

Neurodegenerative diseases such as Alzheimer's and Parkinson's impact millions of people worldwide. Early diagnosis has proven to greatly increase the chances of slowing down the diseases' progression. Correct diagnosis often relies on the analysis of large amounts of patient data, and thus lends itself well to support from machine learning algorithms, which are able to learn from past diagnosis and see clearly through the complex interactions of a patient's symptoms and data. Unfortunately, many contemporary machine learning techniques fail to reveal details about how they reach their conclusions, a property considered fundamental when providing a diagnosis. Here we introduce our Personalisable Clinical Decision Support System (PECLIDES), an algorithmic process formulated to address this specific fault in diagnosis detection. PECLIDES provides a clear insight into the decision-making process leading to a diagnosis, making it a gray box model. Our algorithm enriches the fundamental work of Masheyekhi and Gras in data integration, personal medicine, usability, visualization, and interactivity.

Our decision support system is an operation of translational medicine. It is based on random forests, is personalisable and allows a clear insight into the decision-making process. A well-structured rule set is created and every rule of the decision-making process can be observed by the user (physician). Furthermore, the user has an impact on the creation of the final rule set and the algorithm allows the comparison of different diseases as well as regional differences in the same disease. The algorithm is applicable to various decision problems. In this paper we will evaluate it on diagnosing neurological diseases and therefore refer to the algorithm as PECLIDES Neuro^1^.

## 1. Introduction

The average life expectancy of Europeans increased by 2.9 years in the last decade. People reached an average age of 80.6 in 2013^2^ and there is more than a 50% probability that by 2030, national female life expectancy will break the 90 year barrier (Kontis et al., 2017). But a longer life does not implicate a healthy one. With higher age comes an increased likelihood of chronic diseases. This trend affects the well-being of elderly people and bears huge challenges for society and economics^3^. Computer algorithms and technology can support disease detection and it is hoped that systems like the one presented in this work will become increasingly prevalent as we continue to improve the state-of-the art in predictive medicine.

### 1.1. Neurological Diseases

Alzheimer's and Parkinson's Disease are two of the most common neurodegenerative diseases^3^^,^^4^. In the United States there are currently about 5.5 million patients diagnosed with Alzheimer's Disease (AD) and predictions project this number to grow to about 13.8 million by mid-century. In 2014, official death certificates recorded AD to be the sixth leading cause of death in the US. The average per-person medical payments for services to Alzheimer's patients (or patients with other forms of dementia) older than 65 are three times greater than payments for beneficiaries without these conditions (Association et al., 2017). Over the course of the disease, the structure of afflicted patients' brains changes. A larger amount of so-called plaques and tangles are built by certain proteins, which lead to a loss of connections between nerve cells. This results in the death of nerve cells and a reduced amount of brain tissue. Furthermore, message transmission between neurons is less effective, as certain essential signaling chemicals are missing in the patients' brains^4^ (McKee et al., 1991). Studies have shown that age is the most significant risk factor for AD (Rocca et al., 1991). But there are also genetic factors that can play a role. For example, the Apolipoprotein E (ApoE) gene, in particular one of its three major isoforms is known to be associated with the development of AD (Dawbarn and Allen, 2007; Urdinguio et al., 2009; Criminisi and Shotton, 2013). Different alleles of the gene can indicate higher or lower risk for developing the disease (Corder et al., 1993).

It is estimated that about 1-2% of the world population suffer from Parkinson's Disease (PD). Almost half of the patients develop PD between age 50 and 60 (Mattle and Mumenthaler, 2012). A characteristic of PD is the progressive loss of dopaminergic neurons in the substantia nigra and striatal projections (Urdinguio et al., 2009). Consequently, patients have a lack of dopamine in their brain. While the exact reason for the progressive loss is unknown, its consequences are apparent. The loss of dopamine interrupts patients' ability to smoothly execute planned movements, typified by the three main symptoms of PD: tremor, muscle stiffness, and slowness of movement. Other symptoms include tiredness, pain, depression, and constipation. In the UK, it is estimated that about 145,000 people are currently diagnosed with PD. At present, there is no cure for Parkinson's disease, but treatments can control the symptoms to a certain amount. Drugs, deep brain stimulation, and physical therapies are the most common treatments^3^.

### 1.2. Decision-Making

Making the right decision is one of the key factors of successfully achieving goals in all areas of work and there are numerous ways of finding the right decision. Nevertheless, the basic idea is mostly the same. It is usually a combination of experiences, research results and personal judgement. As the first two components are constantly and rapidly growing, one can imagine that decision-making in general has a great potential to improve over time. But this growth also results in unmanageable amounts of data, which is why we need support systems to help processing them (Podgorelec et al., 2002). The goal of our support system PECLIDES Neuro is to integrate all three mentioned components: experience, research results and personal judgement. Of special importance is the last component. Personal judgement plays a vital role in medical decision-making, but is rarely represented in advanced machine learning processes. Incorporating personal judgement into a decision support system allows a higher degree of interpretability and usability and we believe that data integration is only effective when combined with interpretability and usability.

**Figure 1:**
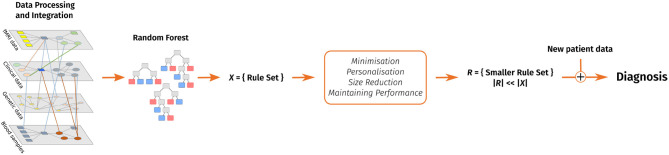
A visual overview of our clinical decision support system. Data pre-processing includes feature extraction. After that, a random forest is trained and converted into a rule set. This rule set is then reduced with the goal of keeping the performance and considering personal preferences in form of favorite features and enables to make a diagnosis on new patient data.

The following sections introduce our decision support system and cover related work, a discussion of the algorithm's design as well as the evaluation on different data sets, various possible applications of the decision support system and future work. Figure 1 shows a graphical overview of our support system.

## 2. Related Work

Supporting medical decisions with current technology is highly discussed in literature. A frequently used technique is the ensemble learning method of random forests. Random forests are a combination of decision tree predictors and a regression and classification method. Each tree individually votes for the most popular class and their creation depends on the values of a random vector which is sampled independently but with the same distribution for all trees in the forest. In general, random forests are robust against over-fitting, run efficiently on large data and handle heterogeneous data well (Breiman, 2001; Louppe, 2014). Moreover, as random forests are based on decision trees, they can be used to explicitly and understandably describe a decision-making process.

### 2.1. Rule Extraction From Random Forests

One disadvantage of random forests is that they can grow very big and subsequently, more unclear. Nonetheless, by extracting rules from a built forest, one can gain an insight into the decision-making process. Mashayekhi and Gras (Mashayekhi and Gras, 2015) introduced two methods called RF+HC and RF+HC_CMPR which allow to extract a rule set from random forests. The main idea is to radically reduce the number of rules and therefore increase the comprehensibility of the underlying model. The rule extraction can be seen as an optimization problem and finding the best rule set is an NP-hard problem (Mashayekhi and Gras, 2015). This is why heuristic approaches are needed to feasibly extract an appropriate subset of rules from the whole rule set.

The algorithm proposed by Masheyekhi and Gras consists of four steps. First, the random forest is generated and all rules are extracted into a rule set. Second, a score is defined for each extracted rule. For the RF+HC method they used Equation (1), wherein *cc* stands for correct classification and is the number of covered training samples that are classified correctly. The variable *ic* refers to the incorrect classification, the number of incorrectly classified training samples, and *k* is a predefined positive constant value. Mashayekhi and Gras proposed to set *k* = 4.

(1) $$\begin{array}{l} score_{1}=\frac{cc-ic}{cc+ic}+\frac{cc}{ic+k} \end{array}$$

This score leads to the elimination of noisy rules and the maintenance of rules with higher accuracy. In the third step of the algorithm a final rule set is generated wherein the probability of selecting a rule is proportional to its score from Equation (1). The probability of selecting a rule is hereby proportional to its score. Finally, the extracted rule set is applied on the test data set to evaluate its performance. The average rule set size after applying the RF+HC algorithm is 0.6% of the original rule set, while the accuracy generally only decreases by a couple of percent (Mashayekhi and Gras, 2015).

Masheyekhi and Gras' second method, called RF+HC_CMPR, modifies *score*_1_ with an addend that considers the length of a given rule:

(2) $$\begin{array}{l} \begin{array}{c} score_{2}=\frac{cc-ic}{cc+ic}+\frac{cc}{ic+k}+\frac{cc}{rl}=score_{1}+\frac{cc}{rl} \end{array} \end{array}$$

Hereby *rl* refers to the length of the rule. This way a shorter rule adds a higher number to the original score than a long rule. The purpose is to favor shorter rules, since they are more transparent and understandable than longer rules. Mashayekhi and Gras applied their two algorithms to several different data sets and compared them to CRF and the standard random forest methods. CRF is a method introduced by Liu et al. (Liu et al., 2011, 2012) which combines rule extraction and feature selection. On average, both RF+HC and RF+HC_CMPR resulted in almost the same accuracy as the CRF method. Furthermore, on average over all data sets, the three methods obtained 96% of the random forest accuracy. Moreover RF+HC and RF+HC_CMPR resulted in a clearly smaller number of rules. On average, the size of the extracted rule set is 0.6% of the original random forest. In comparison, the total number of rules in CRF is 11.66% of the size of the original rule set (Mashayekhi and Gras, 2015).

### 2.2. Shrinking Random Forests

Since random forests can grow very big, it is useful to consider different ways of shrinking a random forest while maintaining its prediction accuracy. To minimize the size of a random forest, one has to decide when and which trees can be eliminated. Zhang and Wang (Zhang and Wang, 2009) presented three different measures to determine the importance of a tree. (1) A tree is not necessary if its removal from the forest has the least impact on the overall prediction accuracy. Furthermore, a tree can be removed if it is highly similar to other trees in the forest. This similarity can be either measured as (2) an average similarity to all other trees or (3) a pairwise similarity.

To get the tree with the least impact on the forest (1), firstly the prediction *P*_*F*_ of the whole forest is calculated. Then, for each tree *T* in the forest *F* the prediction *F*_−*T*_ of the forest without the tree *T* is determined. Lastly, the tree that leads to the smallest difference in prediction accuracy (see Equation 3) can be removed.

(3) $$\begin{array}{l} \delta_{-T}=P_{F}-P_{F_{-T}} \end{array}$$

The similarity between two trees can be defined by the correlation between their predicted outcomes. The average similarity (2) can be calculated as following:

(4) $$\begin{array}{l} \rho_{T}=\frac{1}{N_{F}-1}\underset{t\in F,t\neq T}{\sum }cor_{t,T} \end{array}$$

where *N*_*F*_ is the number of trees in the forest and *cor*_*t,T*_ is the correlation between two trees *t* and *T*. The tree T with the highest ρ_*T*_ has the highest similarity to the rest of the forest and can therefore be eliminated.

The highest pairwise similarity (3) is measured by the correlation of the accuracy of two trees. Firstly, a weight *w*_*T*_ is introduced for every tree T and set to 1. Subsequently, one is searching for the two trees *T*_*s*1_ and *T*_*s*2_, which are most similar. Afterwards, the average of similarity ρ_*s*1_ and ρ_*s*2_ for those two trees is calculated. The tree *T*^*rs*^ with higher ρ can then be removed. Finally, the following weights are calculated:

(5) $$w_{t}^{\prime }=w_{t}+\frac{cor\left(T^{rs},t\right))}{\rho T^{rs}}*\left(N_{F}-1\right),t\in F_{-T^{rs}}$$

As a last step, it is important to select the optimal size for the sub-forest. Zhang and Wang proposed to define a performance trajectory *h*(*i*), *i* = 1, …, *N*_*f*_ − 1 of a sub-forest of *i* trees, where *N*_*F*_ is the size of the original random forest. The optimal size can then be selected by maximizing *h*(*i*) over *i* = 1, …, *N*_*F*_ − 1 (Zhang and Wang, 2009).

Zhang and Wang showed on real data sets that a shrunken random forest can sometimes even outperform the original one and often achieves a very similar accuracy to the original random forest (Zhang and Wang, 2009).

## 3. PECLIDES Neuro

Our implemented clinical decision support system is based on the machine learning technique of random forests. A graphical overview of the algorithm is shown in Figure 1. The first step of the algorithm is to generate a random forest from a given data set and subsequently extract rules from it. The usage of the *RandomForestClassifier*^5^ in Python allows the variation of several parameters relevant to our task. In this project, we focused on the calibration of the number of trees in the forest, the function to measure the quality of a split, the maximum number of features to consider when looking for the best split, and the maximum depth of a tree.

To start the training process of the random forest, the data set was separated into a training and a test set. One approach we used is to generate a train-test split which takes a certain percent of the whole data set (for example 30%) aside for the test set and trains the model on the remaining samples (70%). Using 10-fold cross-validation, the performance on the data set can be measured while reducing the risk of over-fitting.

### 3.1. Extracting Rule Set From Random Forest

**Figure 2:**
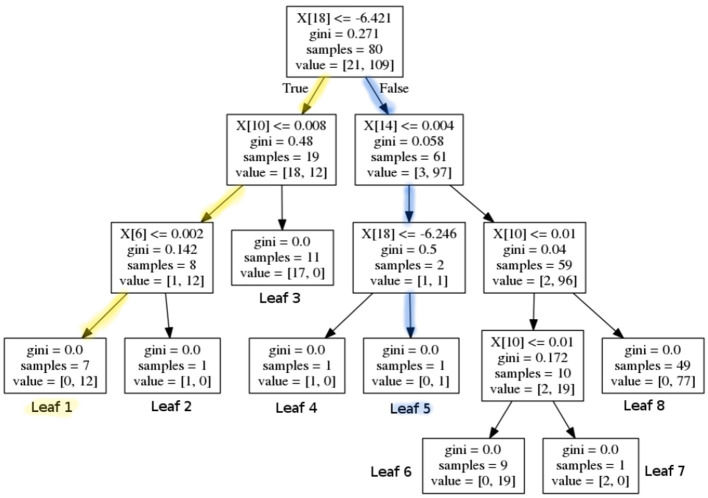
A sample decision tree that would lead to eight rules; the yellow (outer left) and blue path (leading to leaf 5) show two exemplary rules that would be extracted from this decision tree.

The next step after the creation of the random forest is to extract rules from the latter. These rules will then build the core of the decision support system. This can be done by iterating through each tree in the forest and extracting each branch. Figure 2 shows an exemplary decision tree. One branch represents one rule, as it determines the decision process from the tree's root to one leaf. Thus, the displayed tree would lead to eight rules, since it has eight leaves. In each node of the tree, the base logic is, “If *feature*_*X*_ is smaller than Y, then left, else right.” Therefore, for each node the following three values are important: (1) the *feature*_*X*_ that is considered, (2) the value Y that it is checked against and (3) whether the feature has to be smaller or greater than that value. Finally, the outcome of a branch has to be stored. This outcome can be the decision whether a person has a certain disease or not. In our case the trees' leaves determine whether a person is predicted to have Alzheimer's or Parkinson's Disease or whether the person is predicted to be healthy. All rules of all branches of all trees can then be stored in a set that represents the whole rule set of the random forest.

Each branch in a decision tree can be represented by a disjunctive normal form (DNF) where all queries of all nodes are connected with “and.” So the rule from the yellow (outer left) path in Figure 2 can be represented by the following DNF:

(6) $$\begin{array}{c} (feature_{18}<=-6.421\;\wedge \;feature_{10}<=0.008\\ \wedge feature_{6}<=0.002) \end{array}$$

### 3.2. Minimizing the Rule Set

As the number of trees and their depths determine the number and the length of the rules, the exact size of the rule set varies from application to application. But in general, the goal is to keep the rule set small. Figure 2 shows an exemplary decision tree. One can see that feature 18 is checked twice in the blue branch that leads to leaf five. The matching DNF is shown in Equation (7). In the tree's root it is checked whether feature 18 is greater than −6.421 and two layers below it is checked whether feature 18 is greater than −6.246.

(7) $$\begin{array}{l} (feature_{18}>-6.421\;\wedge \;feature_{14}<=0.004\\ \wedge feature_{18}>-6.246) \end{array}$$

This example leads to the first step to shorten rules by eliminating redundant queries within a rule, like in our example branch from above. Deleting the first query (whether feature 18 is greater than −6.421) does not change the properties of the rule. The condition that feature 18 has to be greater than −6.246 already implies that condition *feature*_18_ > -6.421 is fulfilled. Therefore this branch (see Equation 7) can be reduced to the following logical statement:

(8) $$\begin{array}{l} (feature_{14}<=0.004\;\wedge feature_{18}>\\ -6.246)\;\Rightarrow Parkinson^{\prime }s\;Disease \end{array}$$

### 3.3. Personalisable Rule Score

As suggested by Mashayekhi and Gras (2015), we also assigned a score to each rule depending on the rule's performance on the training set as well as other components we will define later. The score chosen in this work is an extension of *score*_2_ introduced in their paper (see Equation 2). It considers correct and incorrect classifications as well as the length of a rule and adds a personalisable attribute. This score is then calculated for every rule separately. The consideration of a personal preference for features enables the rule score to be personalisable and therefore to adjust and influence the support system taking the user's preferences, experience, and expertise into account. The physician can nominate a desired number of features that shall be preferred during the minimization process and are consequently more likely to be kept in the rule set. This personalization can lead to a more suitable support system for physicians and therefore increase the trust in the system and maybe also the willingness to utilize it. By defining the importance of features, it is also possible to take regional differences or patient specific knowledge and data into account.

If a rule does not contain a nominated favorite feature, the score will be the same as rule *score*_2_ (see Equation 2). But if the rule contains favorite features, another value is added to increase the score. This way rules that contain a favorite feature get a higher score than others, even if the performance is the same. Since rules with a low score are eliminated first, this leads to the fact that rules which contain favorite features are less likely to be deleted from the rule set.

Equation (9) shows the new rule score. If several features are defined as preferred ones, than there will be a ranking among them. The features are a parameter list where the first list element is the most preferred feature and the last list element the least preferred one. The score will be the highest, if a rule contains all preferred features (considering the same performance). We decided to use a linear function to calculate the additional score points. The new personalisable rule score is shown in Equation (9). For each occurrence of favorite features in the rule, another addend will be added that will increase the score.

(9) $$\begin{array}{l} \begin{array}{c} score_{pers}=\frac{cc-ic}{cc+ic}+\frac{cc}{ic+k}+\frac{cc}{rl}+\frac{x}{\left(i+2\right)} \end{array} \end{array}$$

Here *i* refers to the feature's index in the list and *x* is a constant positive value that can be customized. So the first feature (index 0) increases the score by $\frac{x}{0+2}=\frac{x}{2}$, whereas the second feature (index 1) increases the rule by $\frac{x}{3}$ and so on. This way the favorite features are ranked and depending on how many and which of those features are considered in one rule, the rule gets a higher or lower score. The parameters *ic*, *cc*, and *k* are the same as suggested by Mashayekhi and Gras (2015). *cc* and *ic* stand for correct and incorrect classification, respectively and *k* is a positive constant value. The usage of *k* = 4 was suggested by Mashayekhi and Gras. The constant *x* can be adjusted depending on how much impact one wants the preferences to have on the whole score. In the following we will use *x* = 40 if not otherwise stated. It is important to determine the impact of the personalization on the algorithm within a reasonable range. We do not want to overrule the classification of the random forest completely, but only enhance the underlying decision-making process with the personalisable factors. For this purpose, the weight of the personalization can be regularized with the factor *x* in Equation (9). We decided to set the increase of the rule score to up to one third of the maximum original score, for the first preferred feature. So in the case discussed here, the maximum rule score without preferences for features was 60 and therefore we used *x* = 40, which leads to an increase of $\frac{40}{2}=20$ for the first feature. The goal is to ensure an impact of the personalization while making sure that the original algorithm is not overruled.

To allow the physician to also put less value on certain features, the personalisable rule score (see Equation 9) can be extended by another addend that decreases the rule score if the rule contains certain features. This way the user can for example express that there is less trust in certain features of the data set. This might be useful in case a measurement went wrong or if for a certain feature several data points are missing. Therefore we propose using the last addend of Equation (9) again, but this time subtracting it from the rule score if the rule contains features that shall be used with caution.

After calculating the score for each rule, the rule set can be minimized to the best performing rules with the most occurrences of preferred features. For that, one can either define a certain percentage of the rule set that shall be deleted or kept, or a threshold to what minimum performance the rule set should be reduced. The latter holds the challenge that the performance of a rule set is not proportional to its size. It is more straightforward to reduce the rule set to a certain percentage, like 10% of the original rule set's size and then analyse the performance of the smaller and more accessible rule set. In addition to the prediction yielded by inspecting the small rule set, it is possible to see which features are monitored and which thresholds are important. This can lead to information about the disease, its characteristics, and possible factors for diagnoses.

### 3.4. Evaluation on Different Data Sets

We used a variety of data sets such as Alzheimer's Disease Neuroimaging Initiative (ADNI, 2017), PROPAG-AGEING^6^, a data set of spiral drawings (Par, 2014) and biomedical voice measurements (Little et al., 2007; Par, 2007). The data are partly a complex collection of different data types and from different institutions in Europe like PROPAG-AGEING.

The ADNI data set contains different types of data, including PET images, MR images and clinical data. Initially, numerical data with information about age, ethical background, gender, and numerous numeric test results were used. The data set consists of 3,445 samples from Alzheimer's patients and healthy subjects. The random forest achieved an accuracy of 97%, a sensitivity of 96% and a specificity of 97.4%. The rule set extracted from the random forest contains 1,447 rules and the performance is strongly dependant on the way the rule set is evaluated.

After deleting the 500 weakest rules of the rule set without any feature preference, the accuracy was still high with 97%, the sensitivity is 96.5% and the specificity 97.2%. Going down to half the rule size with 724 rules resulted in an accuracy of 94.7%, a sensitivity of 86.76% and a specificity of 98.11%. In general, shrinking the rule set based on our score does not have a big impact on the performance.

ADNI also provides three-dimensional *T*_1_-weighted magnetic resonance imaging (MRI) for developing and testing analysis techniques for extracting structural endpoints. To ease the utilization of the MR Images, standardized analysis sets of data comprising scans that met minimum quality control requirements were created within ADNI. In this work, samples from 1-year completers were used, including images from subjects who had 6- and 12-month scans (Wyman et al., 2013). The images typically consist of 256 × 256 × 170 voxels with a voxel size of 1 × 1 × 1.2*mm* (Gaser et al., 2013). 27 scans from Alzheimer's patients and 25 scans from healthy subjects were used.

**Figure 3:**
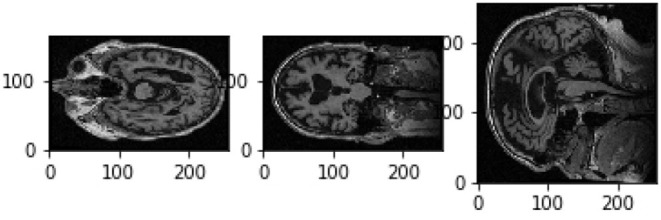
Three slices of a 3D MR image; with sagittal, coronal, and axial plane.

In order to train a random forest, features have to be extracted from the MR images. We used Python and the libraries *nipy*^7^ and *nilearn*^8^ to process the images. Figure 3 shows the three middle slices of an exemplary MR image (sagittal, coronal and axial plane).

**Figure 4:**
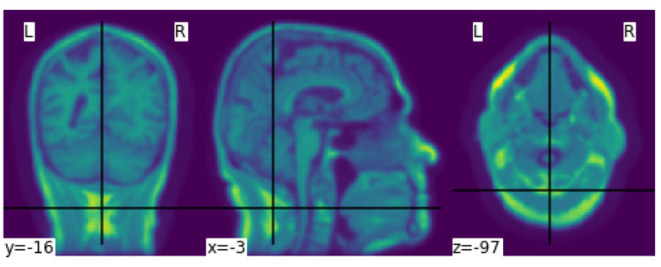
Smoothed MR image with Gaussian filter.

The features extracted from the MR images for this work include first order and second order descriptors (Despotović et al., 2015). As the pre-processing step, a Gaussian filter was applied to the images (see Figure 4). The filter was applied along the three first dimensions of the image^9^. From these, pre-processed arrays features were extracted. The first order descriptors include the sum, the mean and the maximum of all voxel values, as well as mean, sum and maximum values of the middle slices of all three dimensions. Exemplary slices are shown in Figure 3. A color level histogram was used to extract the most frequent voxel value in the MR image. The value 0 was excluded form the histogram, as the background is represented by 0 and should not be considered.

**Figure 5:**
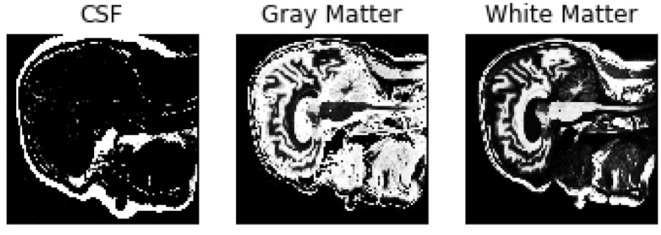
Probabilities of the different brain tissues CSF, gray matter, and white matter.

To extract second order features, the probability of the different brain tissues were determined. The three types of tissue are cerebrospinal fluid (CSF), gray and white matter. Figure 5 shows the probabilities for the different tissue types in an exemplary slice of an MR image. The segmentation was performed using the python library *dipy*^10^, its class *TissueClassifierHMRF* and the Markov Random Fields modeling approach. The latter is frequently used in literature. An example would be Held et al. who described a fully automated 3D segmentation technique for MR images (Held et al., 1997). The maximum a-posteriori Markov Random Field approach uses iterative conditional models and expectation maximization to estimate the parameters^11^. After the segmentation, more features can be extracted depending on the tissue type. The sum of all voxel values separated by tissue type, as well as the maximum of the sum of the inner arrays were calculated. All features were based on the smoothed images.

These features were then used to implement a random forest. Using train test split lead to an accuracy of 99.38%, a sensitivity of 99%, and a specificity of 100% on the test set. Extracting a rule set from a parameter pruned random forest leads to a rule set of 213 rules with an accuracy, sensitivity and specificity of 100% each. The first reduction step that eliminates queries within singular rules (see section 3.2) does not change the outcome. Interestingly, even one rule alone could predict the correct outcome for each data sample. This feature was the maximum of the sum of the sum of the smoothed images. Consequently, this feature defines a clear threshold between AD patients and the healthy control group in this data set, which may be due to the small data set and has to be tested on larger data sets again. But it might be an interesting indicator and be worth looking into further. This shows that PECLIDES can identify possibly important features in a diagnosis process.

**Figure 6:**
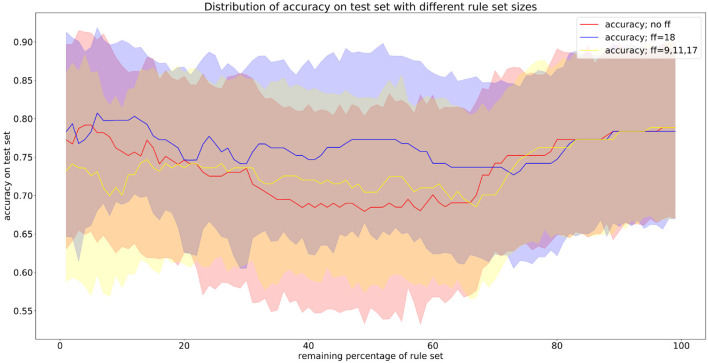
Results from applying PECLIDES Neuro to the Speech Data Set. The line plots show accuracies depending on different rule set sizes. The x-axis indicates the percentage of rules from the original rule set, which remained in the reduced rule set. The different colors refer to different choices of favored features (ff). 100% of the rule set for this data set refers to 358 rules. The error ranges are based on 10-fold cross-validation.

Figure 6 shows the performance of three exemplary rule sets trained on the Speech Data Set, which consists of biomedical voice measurements. The list of features can be found in Table 1. The evaluation shown in Figure 6 was performed on the test set, using 10-fold cross-validation. The red line represents the accuracy of the rule set without choosing any preferred features in the reduction process. We will refer to this scenario as the baseline. The x-axis indicates the size of the minimized rule set in respect to the original rule set. The yellow line shows the accuracy of the different rule sets while preferring features 9, 11 and 17 during the reduction process. For the blue line, feature 18 was favored, which is a non-linear measure of fundamental frequency variation. Feature 18 is the most commonly represented feature in the original random forest. Features 9, 11, and 17 on the other hand are represented little in the original random forest. We chose those features, assuming that the frequency of features in a random forest hold some information about their importance in the decision-making process. The plot shows clearly that the reduction of the rule set still allows to maintain high performance in all three cases. The accuracy of the original rule set (100% on the x-axis) lies at about 0.79 for all three rule sets. The slight difference at the complete rule set is assumed to be due to cross-validation and therefore the different choices for train and test sets. We can see that the rule sets' performances vary with the selection of favorite features. When preferring features 9, 11 and 17 in the reduction process the performance of the rule set is quite similar to the one where no preferred features were set. This leads to the conclusion that those features do not have a particularly high impact on the diagnosis of Parkinson's Disease (PD). When preferably keeping rules with feature 18 in the rule set the accuracy is almost constantly higher than of the baseline (red). This indicates that feature 18 is a better indicator for diagnosing PD than other features. Here we also applied leave-one-subject-out cross-validation to further assess possible over-fitting and achieved an average accuracy over all subjects of 80%, which matches the accuracy of the 10-fold cross-validation mentioned above and shown in Figure 6.

**Table 1:** Feature explanation of Speech Data Set (Little et al., 2007; Par, 2007).

**Feature index**	**Feature name**	**Interpretation**
0	MDVP:Fo(Hz)	Average vocal fundamental frequency
1	MDVP:Fhi(Hz)	Maximum vocal fundamental frequency
2	MDVP:Flo(Hz)	Minimum vocal fundamental frequency
3	MDVP:Jitter(%)	Measure of variation in fundamental frequency
4	MDVP:Jitter(Abs)	Measure of variation in fundamental frequency
5	MDVP:RAP	Measure of variation in fundamental frequency
6	MDVP:PPQ	Measure of variation in fundamental frequency
7	Jitter:DDP	Measure of variation in fundamental frequency
8	MDVP:Shimmer	Measure of variation in amplitude
9	MDVP:Shimmer(dB)	Measure of variation in amplitude
10	Shimmer:APQ3	Measure of variation in amplitude
11	Shimmer:APQ5	Measure of variation in amplitude
12	MDVP:APQ	Measure of variation in amplitude
13	Shimmer:DDA	Measure of variation in amplitude
14	NHR	Measure of ratio of noise to tonal components in the voice
15	HNR	Measure of ratio of noise to tonal components in the voice
16	RPDE	Non-linear dynamical complexity measure
17	DFA	Signal fractal scaling exponent
18	spread1	Non-linear measure of fundamental frequency variation
19	spread2	Non-linear measure of fundamental frequency variation
20	D2	Non-linear dynamical complexity measure
21	PPE	Non-linear measure of fundamental frequency variation

### 3.5. Comparison of Models

**Figure 7:**
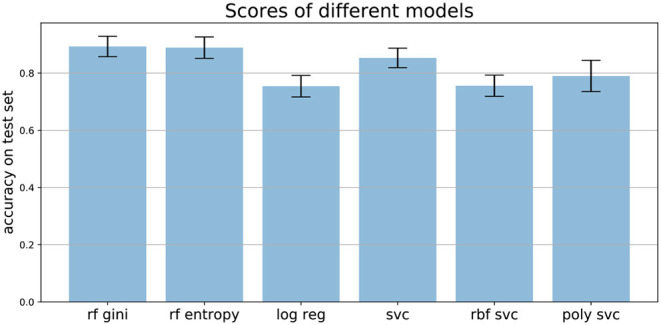
Mean accuracies on test set of different models applied to the Speech Data Set (see section 3.4 for more information on data set). Error bars come from 100 independently trained models each. rf gini, Random forest with Gini impurity; rf entropy, random forest with entropy impurity; log reg, logistic regression; svc, support vector machine with linear kernel; rbf svc, support vector machine with Gaussian kernel; poly svc, support vector machine with polynomial kernel.

We compared the performance of random forests to other machine learning techniques, like logistic regression and different support vector machines, as well as comparing the two common impurity measures: Gini and Entropy. Figure 7 shows the mean accuracies of different models trained on the Speech Data Set. The error bars indicate the accuracies of 100 independently trained models each. The first two bars indicate the accuracies of random forests trained with Gini impurity and Entropy (information gain), respectively. The third bar shows the performance of a logistic regression with L1 penalty and the Saga solver, which uses Stochastic Average Gradient descent^12^. The three most right bars show results of three support vector machines, trained with different kernels: linear (svc), Gaussian (rbf svc) and polynomial (poly svc). The random forests outperform the other models on this data set and the difference in performance of the different impurity measures, which measure the quality of split within the trees, is minor. This matches literature, since it has been found that the Gini criteria and the entropy criteria only disagree in about 2% of the cases (Raileanu and Stoffel, 2004).

### 3.6. Handling Missing Values

Our rule based algorithm depends on the previous implementation of a random forest. The random forest itself does not handle missing values, but Python's *sklearn* (Pedregosa et al., 2011) package provides a class called *Imputer*^13^ that can handle missing values and replace them with either the mean, the median, or the most frequent value in the respective column. This way the support system can handle missing values and incomplete data samples do not have to be deleted. This was for example applied in the PROPAG-AGEING data set, as some values were missing in a few data samples. One could also take this into account during the reduction step of the rule set by not choosing those less reliable features as preferred ones or even penalizing rules which contain less reliable features. This shows another advantage of the fact that the support system is personalisable.

### 3.7. Graphical User Interface

**Figure 8:**
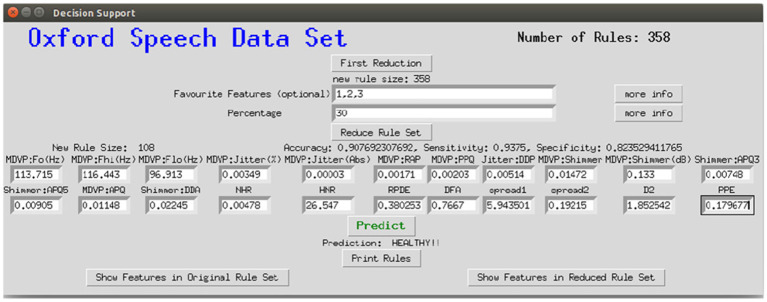
Graphical interface for the clinical decision support system, here applied to the Speech Data Set.

**Figure 9:**
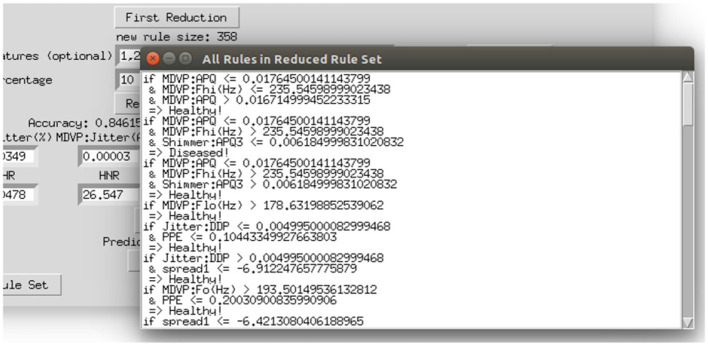
Displaying all rules that remained in the reduced rule set.

**Figure 10:**
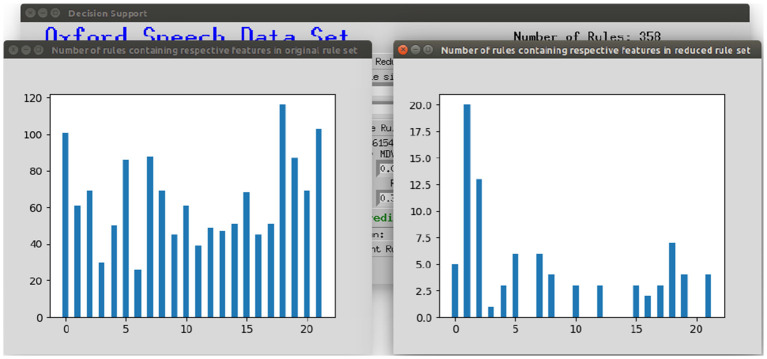
Displaying the number of rules containing the respective feature in the original rule set and the current reduced rule set.

To make the interaction with the decision support system approachable and straightforward, a user interface was implemented. Figure 8 shows the provided interface. The first rule set that was extracted from the whole random forest was already created beforehand. The name of the data set and the number of rules of this original rule set are stated on top of the window. As shown beneath, the first reduction step can be performed by clicking on the button *First Reduction*. This does not change the number of rules but eliminates redundant queries within one rule (see section 3.2 for more information). The new size of the rule set is then stated under the button. In the next step, optionally favorite features can be named (1, 2, and 3 in the example figure) and the percentage to which the rule set shall be reduced (here: 30%). The new size as well as accuracy, sensitivity and specificity will then be calculated and displayed after the button *Reduce Rule Set* is clicked. To make a new prediction, a value has to be filled in for each feature and with the button *Predict* a prediction is calculated and displayed (here: healthy). The button *more info* opens a message box with more information about what to fill in the entry boxes. The button *Print Rules* shows all rules within the current reduced rule set in form of if statements (see Figure 9). The two buttons on the bottom of the graphical user interface show bar charts with the number of rules containing each feature in the original rule set and the current reduced one (see Figure 10). These two properties allow an insight into the decision-making process as well as the impact of different features.

### 3.8. Advanced Usage of the PECLIDES Library

In general, data integration techniques often require additional hypotheses on missing or low quality data, causality pathways and dependability factors, to resolve contrasting phenotypic patterns, leading to different diagnoses or therapies (for instance different drug dosages). Thus, confidence in the diagnosis requires the identification of a trust-region, which could suggest the acquisition of further evidences or the re-analysis of the cost-benefit function in light of therapeutic urgency (time).

White box modeling approaches are hypothesis- or model-driven, while black box models are based entirely on data. The combination of the two, forms the so called gray box model approach. Gray box modeling combines a partial theoretical structure with data to complete the model. A meaningful way to provide the black box model with capabilities of considering and testing different hypothesis, is to use an interactive visualization approach. The part of the brain that processes visual information is extremely well-developed during evolution while reading large volume of numbers is a less evolved skill, which is only a few thousand years old (Dehaene, 2005). Therefore, we decided to use a graphical user interface to improve our ability to tune the gray box model and integrate both hypotheses (for example on missing values or experience biases) and data to increase confidence in the diagnosis and a large region of trust.

A more advanced interactive visualization tool could facilitate the task of integrating artificial intelligence and machine learning based data analysis, using techniques such as random forests or neural networks with reasoning and hypothesis on causality trajectories, missing values, confounding factors, sources of errors, different weights on the data integration to generate a robust and friendly Clinical Decision Support System (CDSS). The graphical user interface will empower the gray box model by adding interacting hypothesis-testing visualization to the already developed machine learning tool, resulting in an improved interpretability, explainability and participation.

## 4. Conclusions

The algorithm introduced in this work can be used as a clinical decision support system. It assists in the analysis of clinical data, integration of different data type, and finally, provision of a diagnosis. The focus of this work is thereby on neurological diseases like Alzheimer's and Parkinson's Disease. The goal is to make machine learning algorithms more transparent and accessible while ensuring a high performance. Therefore, we provide a gray model approach, which is especially useful in clinical contexts since a comprehensible decision-making process increases the trust in the diagnosis and can reveal new information about diseases. The PECLIDES toolbox includes the option of personalizing and adjusting the treatment of parameters within the algorithm. This leads to more insight into features used by the algorithm, their importance and informative value.

The algorithm can be divided into three major steps (1) Firstly, a random forest is created that builds the foundation of the algorithm. (2) Secondly, a set of rules is extracted from the random forest. (3) And finally, this rule set is reduced using different algorithms. The third step includes the personalisable aspects, where preferences for important features can be set. Figure 1 shows a graphical overview of the algorithm.

**Figure 11:**
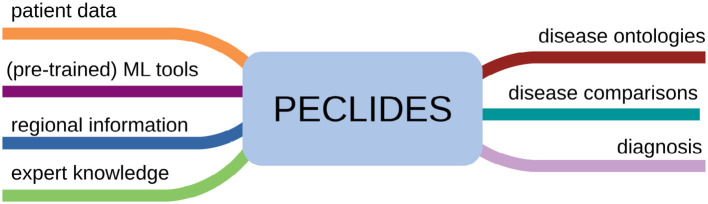
Overview of possible applications of the PECLIDES library. On the left several different inputs to the algorithm are shown. Patient data, (pre-trained) machine learning (ML) tools, specific relevant regional information and expert knowledge can be used as input. And the algorithm can provide different outputs, which can be related to disease ontologies or disease comparisons or a diagnosis.

The PECLIDES toolbox has versatile features and applications, as detailed in Figure 11. The algorithm can take different inputs, analyses and combines them, and provides different outputs depending on the application. One fundamental input for making a diagnosis is the new patient data. It will be run through the (pre-trained) machine learning tool, in our case a random forest, and PECLIDES can provide a diagnosis. But since PECLIDES provides a transparent decision-making process the user has the possibility to inspect, change and evaluate the algorithm. Thus, the input can be enriched by providing expert knowledge and regional information, e.g. physicians could add their own rules, prefer certain decision features or neglect others, based on their experience and knowledge. This way, local aspects, ethnic groups, regional lifestyle, environment factors, or common social interactions can be considered. The output is not limited to a diagnosis. Another interesting analysis could be disease comparisons, to evaluate the similarity between different diseases. Therefore, knowledge about the importance of specific factors in two or more diseases are very valuable. By examining the impact of various genes for example, one could reason about the similarity between different diseases or draw conclusions about disease ontologies.

## 5. Future Work

There are several possibilities of combining random forests and neural networks, such as Wang et al. (2018), Zorman et al. (2000), and Humbird et al. (2018). Those two machine learning techniques have many complementary advantages and disadvantages. For example, the knowledge representation of decision trees is mostly comprehensible whereas the decision process of neural networks is hard to understand. On the other hand, decision trees have trouble with noisy data, which is not a big problem for neural networks. So the idea came up to combine these two approaches to benefit from both their advantages (Podgorelec et al., 2002). Zorman et al. introduced an idea of how to combine decision trees and neural networks. First, they generated a decision tree which is then used to initialize the neural network. Subsequently, the neural network is again converted into a decision tree, which has a better performance than the original one. The resulting decision tree may not have the same performance as the neural network, but it is easier to interpret and comprehend (Zorman et al., 1999, 2000; Podgorelec et al., 2002. This approach could also be used for the clinical decision support system introduced in this work and is a promising approach to make decision support systems more accessible.

Random forests can also be used to initialize deep feed-forward neural networks where the network's structure is determined by the structure of the trees. These so-called “deep jointly informed neural networks” (DJINN) show a warm-start to the neural network training process and result in lower cost and a lower number of user-specified hyper-parameters needed to create the neural network (Humbird et al., 2018). This shows another possibility to combine random forests with neural networks and bring together both methods' advantages. Our on random forests based decision support system could also be used as a foundation for further developments of DJINNs and bears numerous similar possibilities for extensions and systems that could be built on top.

## Data Availability Statement

The source code of the PECLIDES package can be found on GitHub under the following link: https://github.com/tamaramueller/Peclides-Neuro.

The datasets for this study can be found at the following links:ADNI - Alzheimer's disease neuroimaging initiative (2017): http://adni.loni.usc.edu/.

UCI - Parkinson's disease spiral drawings (2014): https://archive.ics.uci.edu/ml/datasets/\Parkinson+Disease+Spiral+Drawings+Using+Digitized+Graphics+Tablet.

UCI - Parkinson's speech data set (2007): http://archive.ics.uci.edu/ml/datasets/Parkinsons.

## Author Contributions

TM and PL developed the theoretical project architecture and ideas and contributed to the final version of the manuscript. TM implemented the software and evaluated it on the data sets. PL supervised the project.

## Conflict of Interest

The authors declare that the research was conducted in the absence of any commercial or financial relationships that could be construed as a potential conflict of interest.

## Footnotes

^2^https://www.parkinsons.org.uk/about-us/media-and-press-office

^3^https://www.parkinsons.org.uk/about-us/media-and-press-office

^4^https://www.alzheimers.org.uk/info/20007/types_of_dementia/2/alzheimers_disease

^5^http://scikit-learn.org/stable/modules/generated/sklearn.ensemble.RandomForestClassifier.html

^6^https://www.propag-ageing.eu/

^7^http://nipy.org/nipy/

^8^https://nilearn.github.io/

^9^http://nilearn.github.io/modules/generated/nilearn.image.smooth_img.html

^10^http://nipy.org/dipy/

^11^https://dipy.org/documentation/1.1.1./reference/dipy.segment/#tissueclassifierhmrf

^12^https://scikit-learn.org/stable/modules/linear_model.html#logistic-regression

^13^https://scikit-learn.org/stable/modules/generated/sklearn.impute.SimpleImputer.html
